# Reactive Oxygen Species Enhance TLR10 Expression in the Human Monocytic Cell Line THP-1

**DOI:** 10.3390/ijms11103769

**Published:** 2010-09-29

**Authors:** Donghee Kim, Yeon Ju Kim, Hyun Sook Koh, Tae Yang Jang, Hyo Eun Park, Jae Young Kim

**Affiliations:** Department of Biological Science, Gachon University of Medicine and Science, Incheon, 406-799, Korea; E-Mails: dh2388@hanmail.net (D.K.); 01191767141@nate.com (Y.J.K.); kohs978@hanmail.net (H.S.K.); jjjangssun@naver.com (T.Y.J.); ispinrosen7@hanmail.net (H.E.P.)

**Keywords:** Toll-like receptor10, reactive oxygen species, hypoxia, monocytes

## Abstract

We investigated TLR10 expression in human monocytes, THP-1 cells, cultured in hypoxia (3% O_2_). Levels of both TLR10 mRNA and protein in THP-1 cells cultured in hypoxia were significantly higher than those cultured in normoxia (20% O_2_). We examined intracellular reactive oxygen species (ROS) content in hypoxic cells, and TLR10 expression in cells treated with hydrogen peroxide (H_2_O_2_), to determine whether the increase in TLR10 expression observed with hypoxia was due to an increase in intracellular ROS levels. We found that the level of intracellular ROS in cells subject to hypoxia was significantly higher than in normoxia. Experiments with ROS synthesis inhibitors revealed that hypoxia induced ROS production is mainly due to NADPH oxidase activity. TLR10 mRNA expression was increased by treatment with H_2_O_2_ at concentrations ranging from 50 to 250 μM. We screened the TLR10 promoter and found putative binding sites for transcription factors (TFs), such as NF-κB, NF-AT and AP-1. Next, we examined TF activities using a luciferase reporter assay. Activities of NF-κB, NF-AT and AP-1 in the cells treated with H_2_O_2_ were significantly higher than in untreated cells. The experiment with TF inhibitors revealed that ROS-induced upregulation of TLR10 expression is mainly due to NF-κB activation. Overall, our results suggest that hypoxia or ROS increase TLR10 expression in human monocytes and the transcriptional activities of NF-κB are involved in this process. Therefore, it is suggested that ROS produced by various exogenous stimuli may play a crucial role in the regulation of expression and function of TLR10 as second messengers.

## 1. Introduction

Toll-like receptors (TLRs), a major class of pathogen recognition receptor, play a crucial role in the induction of innate immunity at the early stage of infection and also in subsequent recruitment of adaptive immunity [1]. When TLRs located on the cell surfaces or in endosomes recognize pathogen associated molecular patterns (PAMPs), the Toll/interleukin-1 receptor (TIR) domain interacts with myeloid differentiation factor 88 (MyD88), common TIR adaptor. This interaction leads to the activation of a downstream signaling pathway and ultimately induces the expression of inflammatory genes via transcriptions factors (TFs) NF-κB or IRF [2].

In mammals, 13 TLRs have been identified which can recognize many structurally different ligands through their distinct extra-domain [3]. In humans, 10 TLRs have been found and their ligands have also been identified except for in the case of TLR10 [2]. Like other TLRs, TLR10 has multiple leucine-rich repeats and TIR domain and TLR10 shares the highest homology with TLR1 and TLR6 [4]. TLR10 can form either a homodimer or a heterodimer with TLR1 or TLR2 and like other TLRs, it transmits an intracellular signal via MyD88 [5]. A recent structural study showed that two TLR10-TIR domain complexes can form a dimer [6].

Local hypoxic conditions can be developed in various inflamed, infected, and diseased tissues due to occlusion of the local blood supply or the inability of local vessel growth to keep pace with the growth and/or infiltration of cells in the affected tissue [7]. In these conditions, macrophages not only increase intracellular levels of H_2_O_2_ but also up-regulate the expression of genes required for macrophage survival, angiogenesis, and recruitment and activation of macrophages and/or other inflammatory cells [7].

Regarding the regulation of TLR expression, it has been reported that hypoxia increases TLR4 expression in human microglia [8] and murine macrophages [9]. Another study showed that hypoxia selectively increased the expressions of TLR2 and TLR6 in murine and human dendritic cells (DCs) [10]. Although not a direct effect of hypoxia, LPS-induced intracellular reactive oxygen species (ROS) production was found to increase TLR4 expression in THP-1 cells [11].

So far, there has been no report of effects of hypoxia or ROS on TLR10 expression except one previous account which stated that hypoxia does not affect TLR10 expression in human DCs, microvascular epithelial cell lines and CaCo-2 cells [10]. In this study, we therefore attempted to examine the effects of hypoxia and ROS on TLR10 expression in human monocytes THP-1.

## 2. Results and Discussion

### 2.1. Hypoxia Enhanced Intracellular ROS Production and TLR10 Expression in THP-1 Cells

**Figure 1:**
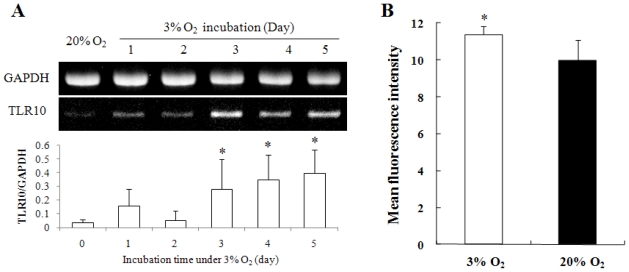
Hypoxia upregulated TLR10 expression in THP-1 cells. (**A**) A representative RT-PCR showing time course expression of TLR10 mRNA in THP-1 cells grown in 3% O_2_ for 5 days. The relative expression of TLR10 mRNA was normalized by GAPDH using densitometric analysis; (**B**) Surface TLR10 expression of THP-1 cells grown in 20% or 3% O_2_ for 3 days was examined by FACS analysis. *P < 0.05.

**Figure 2:**
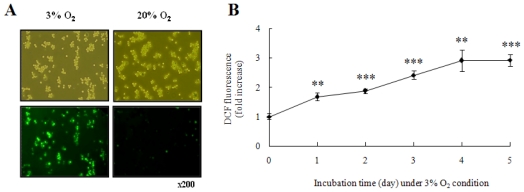
Hypoxia generated intracellular ROS in THP-1 cells. (**A**) Representative fluorescence microscopic images showing the fluorescence of DCF oxidized by intracellular ROS in THP-1 cells grown in 20% or 3% O_2_ for 5 days. After cultivation, the cells were incubated with 10 μM DCFH-DA for 15 min at 37 °C and assessed by fluorescence microscopy; (**B**) Normalized mean fluorescence intensities (MFI) of DCF in THP-1 cells grown in 3% O_2_ for 1–5 days were examined by FACS analysis.

TLR10 expression in human monocytes THP-1 cells cultured in hypoxic conditions (3% O_2_) was investigated. Levels of TLR10 mRNA expression in THP-1 cells cultured for 1 to 5 days in hypoxia were higher than those in normoxia (20% O_2_) (Figure 1A). The level of surface TLR10 expression of cells in hypoxia was also higher than those in normoxia (Figure 1B). Since hypoxia increases intracellular ROS [12,13], and ROS play a role in regulation of gene expression [13,14], we examined intracellular ROS content to determine whether TLR10 expression levels correlated with intracellular ROS levels. Intracellular ROS content in THP-1 cells in hypoxia gradually increased during the cultivation period and was approximately three times higher than in normoxia at day 4 (Figure 2).

**Figure 3:**
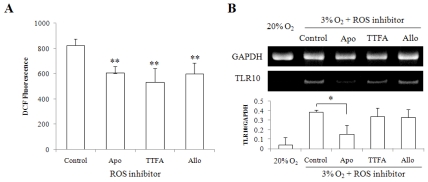
NADPH oxidase inhibitor suppressed intracellular ROS production and TLR10 mRNA expression in THP-1 cells cultured in hypoxia. (**A**) Intracellular ROS production was examined in THP-1 cells grown in 3% O_2_ for 2 days in the presence of three different ROS synthesis inhibitors; Apocynin (Apo, 100 μM), NADPH oxidase inhibitor; TTFA (100 μM), mitochondria electron transport chain complex II inhibitor; Allopurinol (Allo, 100 μM), xanthine oxidase inhibitor. After cultivation, the cells were incubated with 10 μM DCFH-DA for 15 min at 37 °C and assessed by FACS analysis; (**B**) A representative RT-PCR data showing TLR10 expression in THP-1 cells treated with ROS synthesis inhibitor. The cells grown in 3% O_2_ for 3 days in the presence or absence of three different ROS synthesis inhibitors were harvested and examined by RT-PCR analysis. The relative expression of TLR10 was normalized by GAPDH using densitometric analysis. *P < 0.05, **P < 0.01.

There are several sources of ROS in monocytes, including NADPH oxidase, xanthine oxidase and mitochondria [15]. To examine which enzyme systems are involved in ROS generation in hypoxic condition and whether ROS are directly involved in up-regulation of TLR10 expression, we used three different ROS synthesis inhibitors; TTFA, mitochondria electron transport chain complex II inhibitor; Apocynin, NADPH oxidase inhibitor; Allopurinol, xanthine oxidase inhibitor. All three inhibitors were found to be capable of inhibiting intracellular ROS generation in hypoxic conditions (Figure 3A). However, NADPH oxidase inhibitor was found to be only potent to inhibit the TLR10 mRNA expression (Figure 3B). These results indicate that the main enzyme corresponding to the hypoxia-induced up-regulation of TLR10 expression is NADPH oxidase.

### 2.2. Hydrogen Peroxide Enhanced TLR10 Expression in THP-1 Cells in a Concentration-and Time-Dependent Manner

**Figure 4:**
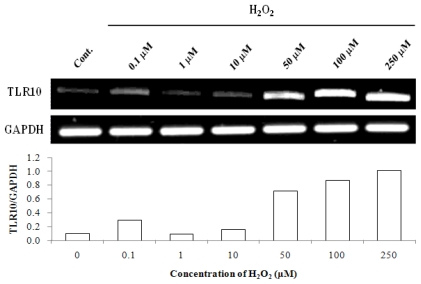
TLR10 mRNA expression in THP-1 cells in the presence of varying concentrations of ROS. A representative RT-PCR experiment showing TLR10 expression in THP-1 cells treated with H_2_O_2_. The cells that had been maintained at 37 °C in 20% O_2_ were treated with varying concentrations of H_2_O_2_ for 1 h and then the cells were harvested and examined by RT-PCR analysis. The relative expression of TLR10 was normalized by GAPDH using densitometric analysis.

**Figure 5:**
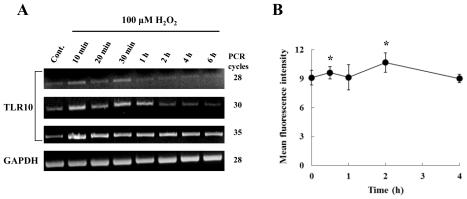
TLR10 expression in THP-1 cells as a function of various incubation times of ROS. (**A**) A representative RT-PCR experiment showing TLR10 expression in THP-1 cells treated with 100 μM H_2_O_2_. The cells that had been maintained at 37 °C in 20% O_2_ were treated with 100 μM H_2_O_2_ for 0 to 6 h as indicated below and then the cells were harvested and examined by RT-PCR analysis; (**B**) Surface TLR10 expression of THP-1 cells treated with 100 μM H_2_O_2_. The cells that had been maintained at 37 °C in 20% O_2_ were treated with 100 μM H_2_O_2_ for 0 to 4 h as indicated below and then the cells were harvested and examined by FACS analysis. *P < 0.05.

Since the levels of intracellular ROS and TLR10 expression were found to be increased in cells in hypoxia, we attempted to find a direct relationship between ROS and TLR10 expression. Since H_2_O_2_ is the most stable and longest lived among ROS [16,17] and is considered an intracellular second messenger as well as an intercellular messenger [18–20], we selected H_2_O_2_ to treat cells to examine the effect of ROS on TLR10 expression in THP-1 cells. Treatment of the cells with H_2_O_2_ at concentrations ranging from 50 to 250 μM significantly enhanced TLR10 mRNA expression (Figure 4). Figure 5A shows the time course of TLR10 mRNA expression in response to 100 μM H_2_O_2_. Expression increased 10 min after incubation and returned to the original level after 1 h. The increase in surface expression was also paralleled by an increase in mRNA level as a function of incubation time (Figure 5B).

### 2.3. NF-κ*B* Inhibitor Suppressed H_2_O_2_-Induced TLR10 Up-Regulation

**Figure 6:**
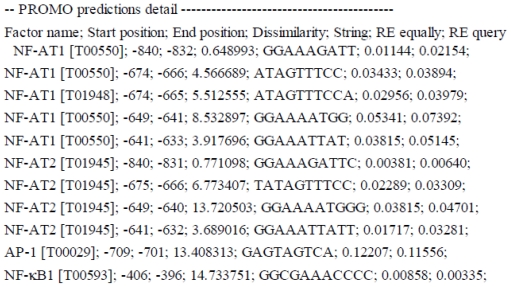
Summary of computer assisted search for the putative binding sites for selected TFs. The putative binding sites were predicted by using Alggen Promo software, V 3.0.2 (http://alggen.lsi.upc.es). Factor name—transcription factor with its TRANSFAC (V 8.3) database accession number; Start and End—start and end positions of putative binding sequences, respectively; Dissimilarity—rate of dissimilarity (%) between the putative and consensus sequences; String—nucleotide sequence of potential binding site; Random Expectation (RE)—expected occurrences of the match in a random sequence of the same length as the query sequence according to the dissimilarity index (RE equally—equi–probability for the four nucleotides and RE query—nucleotide frequencies as in the query sequence).

**Figure 7:**
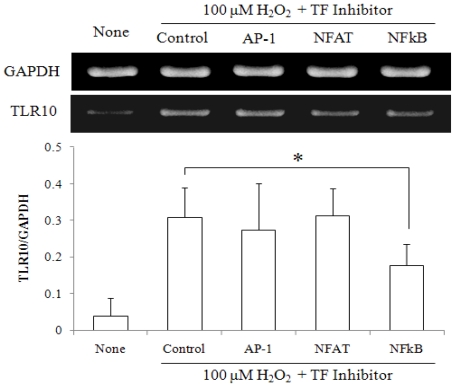
NF κB inhibitor suppressed H_2_O_2_-induced up-regulation of TLR10 expression. A representative RT-PCR data showing TLR10 expression in THP-1 cells were treated with TF inhibitors for 30 min or 1 h before treatment with 100 μM H_2_O_2_. AP-1, NF-AT and NF- κB were blocked with curcumin (Sigma, 10 μM, preincubation for 30 min), a cell-permeable NF-AT inhibitor peptide (Calbiochem, 4 μM, for 1 h) and quinazoline (Enzo Life Sciences Inc., 10 μM, for 30 min), respectively. The cells that had been maintained at 37 °C in 20% O_2_ in the presence or absence of the inhibitors were treated with 100 μM H_2_O_2_ for 1 h, harvested and then examined by RT-PCR analysis. The relative expression of TLR10 was normalized by GAPDH using densitometric analysis. *P < 0.05.

**Figure 8:**
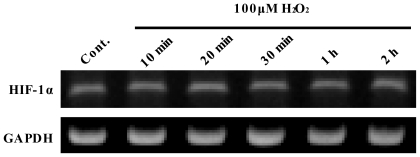
HIF-1 α mRNA expression in THP-1 cells as a function of various incubation times of ROS. A representative RT-PCR experiment showing HIF-1α expression in THP-1 cells treated with 100 μM H_2_O_2_. The cells that had been maintained at 37 °C in 20% O_2_ were treated with 100 μM H_2_O_2_ for 0 to 2 h as indicated below and then the cells were harvested and examined by RT-PCR.

To determine whether TFs, such as NF-κB, NF-AT and AP-1, which can be activated in response to various stresses [21], are involved in the increase in TLR10 mRNA expression by H_2_O_2_, we screened the TLR10 promoter to identify putative binding sites for TFs using a binding site prediction program. The total numbers of conserved binding sites for NF-κB, NF-AT and AP-1 within the TLR10 promoter region (defined as 1000 bp upstream of the transcription start site) were 1, 9, and 1, respectively (Figure 6). The luciferase-reporter assay revealed that treatment of the cells with 100 μM H_2_O_2_ enhanced the activities of NF-κB, NF-AT, and AP-1 (Table 1). To examine which TFs are involved in ROS-induced up-regulation of TLR10 expression, we used three different TF inhibitors. As shown in Figure 7, NF-κB inhibitor significantly inhibited the H_2_O_2_-induced up-regulation of TLR10 mRNA expression but other inhibitors did not. Therefore, the increase in TLR10 expression of THP-1 cells by H_2_O_2_ seems to be mainly due to NF-κB activation. Since hypoxia-inducible factor (HIF-1) has been known to play a central role in regulation of cellular responses to hypoxia [22,23] and in regulation of the innate immune function of phagocytes [24], we examined the possible involvement of HIF-1 in the increase in TLR10 mRNA expression. An HIF-1 binding site search revealed no binding site up to 3000 bp upstream of the transcription start site. Expression levels of HIF-1α mRNA in cells treated with 100 μM H_2_O_2_ were not changed (Figure 8).

### 2.4. Discussion

To our knowledge, this is the first study demonstrating up-regulation of TLR10 expression in human monocytes cultured in hypoxia or in the presence of H_2_O_2_. TLR10 is the only orphan receptor among the human TLRs. One major hindrance to TLR10 study is the absence of its mouse homologue. Therefore, studying the expression of TLR10 in human immune cells is essential. Changes of expression patterns in response to certain stimuli may help us predict its possible ligand(s). TLR10 expression has been known to be mainly restricted to B cells and plasmacytoid DCs [2,5]. However, the present study showed TLR10 expression in human monocytic cell line, THP-1 which is in agreement with a previous report [4]. We also found surface expression of TLR10 in human primary monocytes (data not shown).

Although a natural ligand for TLR10 has not been identified so far, structural or functional studies have suggested, that like other TLRs, it can transfer intracellular activation signals through MyD88 [5,6]. In addition, a study on TLR10 polymorphism with asthmatic patients suggested that TLR10 may be involved in the recognition of airborne pathogens or airborne allergens [25]. It has also been reported that some haplotype of TLR10 is associated with increase in the risk of nasopharyngeal carcinoma [26]. In mammals, TLR1, 6, and 10 genes are tandemly arranged and seem to have arisen from duplication event [27,28]. Studies on single nucleotide polymorphisms in the TLR1-6-10 gene cluster have suggested that mutations within this cluster may be associated with a risk of asthma [25], prostate cancer [29] or non-Hodgkin lymphoma [30].

Macrophages play a critical role in certain circumstances such as bacterial infection, tissue injury, inflammatory disease, and tumor formation. The genes that are up-regulated in these circumstances largely overlap with those in hypoxia, which inevitably develops in these circumstances [7]. Based on this relationship, we speculate that TLR10, whose expression level in THP-1 cells was found to be enhanced by hypoxia, may play a role in immune responses of human monocytes to bacterial infection, tissue injury, inflammatory disease, and tumor formation.

When cells are exposed to hypoxic conditions they produce and use ROS as second messengers in response to hypoxia [12]. Macrophages also produce ROS in response to hypoxia, which can affect the expressions of various genes [7]. Genes that are increased by hypoxia mainly include those involved in cell survival, angiogenesis, and activation and recruitment of leukocytes, including macrophages themselves [7]. ROS can be produced by pro-inflammatory stimuli including TNF-α, IL-1β, and LPS [31,32]. In turn, ROS can induce inflammatory responses via NF-κB or AP-1 activation [33]. It has been well known that hypoxia [34] and ROS [35] play a role in the activation of NF-κB, AP-1, and HIF-1 that contribute to gene regulation in inflammation. Intermittent hypoxia has been shown to activate NF-AT as well as NF-κB, AP-1, and HIF-1 [36]. The present study revealed that the existence of the putative binding site for NF-κB within the TLR10 promoter region, the activation of NF-κB by H_2_O_2_ and suppression of H_2_O_2_-induced up-regulation of TLR10 expression by NF-κB inhibitor. Considering these results combined with those of previous studies addressing the direct correlation between hypoxia and ROS and the activities of NF-κB, the increases in TLR10 expression of THP-1 cells by hypoxia or exogenous H_2_O_2_ seem to be directly associated with activation of NF-κB.

In hypoxic conditions, HIF plays a major role in TNF and NO production by macrophages [37] and phagocytosis of macrophages [38]. HIF-responsive elements have been known to be located in TLR2 and TLR6 genes and the expression levels of both genes increases in hypoxia in a HIF-1α dependent manner [10]. In the present study, however, no HIF-1 binding site was found within the TLR10 promoter region; therefore, it seems that HIF-1 may not be directly associated with the increase in TLR10 expression by hypoxia or ROS.

## 3. Experimental Section

### 3.1. Reagents

Apocynin (Apo) as an NADPH oxidase inhibitor, allopuronil (Allo) as a xantin oxidase inhibitor, TTFA (2-Thenoyltrifluoroacetone) as a mitochondria electron transport chain complex II inhibitor and hydrogen peroxide (H_2_O_2_) were purchased from Sigma Aldrich Corp. (Sat. Louis, Mo, U.S.). Curcumin as curcuminoid, NF-κB activation inhibitor (6-Amino-4-(4-phenoxyphenylethylamino) quinazoline) and NF-AT Inhibitor were purchased from Sigma, Enzo Life Sciences Inc. (Plymouth Meeting, PA, U.S.) and Calbiochem/EMD Biosciences (San Diego, CA, U.S.), respectively.

### 3.2. Cell Culture

The Human monocytic cell line THP-1 cells were obtained from KCLB (Korean cell line bank, Seoul, Korea) and cultured in RPMI 1640 (Welgene, Daegu, Korea) supplemented with 10% heat-inactivated fetal bovine serum (FBS, Lonza, Basel, Switzerland), 1% Antibiotic-Antimycotic (Invitrogen, Gibco BRL, MD, U.S.), 10 mM HEPES buffer (Invitrogen), 50 μM 2-mercaptoethanol (Invitrogen), THP-1 cells were maintained at 37 °C under 5% CO_2_ in 95% air (20% O_2_). For the low oxygen experiment, cells were maintained in Heracell gas addition incubator (Heraeus Instruments GmbH, Hanau, Germany) with gas mixtures consisting of 3% O_2_, 5% CO_2_, and 92% N_2_ (referred to as 3% O_2_). To measure effects of ROS synthesis inhibitors on the intracellular ROS production in THP-1 cells, we used Apo (100 μM), TTFA (100 μM) or Allo (100 μM). Cells were pretreated with each inhibitor for 30 min before cultivation under 3% O_2_. After 48 h-incubation, intracellular ROS level was measured by FACS analysis. In a separate experiment, after 72 h-incubation under the same conditions mentioned above, TLR10 expression was examined by RT-PCR analysis. To measure effects of TF inhibitors on H_2_O_2_-inducted up-regulation of TLR10 expression in THP-1 cells, 10 μM curcumin (for AP-1), 10 μM QNZ (for NF-κB) were pretreated for 30 min and 4 μM NF-AT inhibitor was pretreated for 1 h before H_2_O_2_ treatment. After 1 h incubation with H_2_O_2_, cells were harvested and TLR10 expression was examined by RT-PCR analysis.

### 3.3. RT-PCR

Total RNA was isolated using the Qiagen RNAeasy kit according to the manufacturer’s instructions (QIAGEN). The concentration and purity of the RNA were determined by OD260/280 reading. The cDNA was synthesized from 2 μg of total RNA with SuperScript III Reverse Transcriptase (Invitrogen) using an Oligo (dT) primer (Invitrogen) at 50 °C for 1 h. PCR amplification was performed using following primer sets: TLR10 5′-ctgggacgaccttttccttatct-3′, 5′-cagagatgggctgagaatgaagt-3′, GAPDH 5′-acagcctcaagatcatcagcaat-3′, 5′-aggaaatgagcttgacaaagtgg-3′, HIF-1α 5′-gtctcgagatgcagccagatctcg-3′, 5′-ggtcagatgatcagagtccaaagc-3′. Thermocycling conditions were 30 sec at 94 °C, 30 sec at 58 °C, and 30 sec at 72 °C for 28–35 cycles preceded by 10 min at 94 °C. PCR products were fractionated by 1.5% agarose gel electrophoresis, stained with ethidium bromide, and visualized with UV light. The bands were quantitated by densitometric analysis using KODAK MI software (Rochester, NY, U.S.).

### 3.4. Measurement of Reactive Oxygen Species

Inside cells, DCFH-DA was cleaved by esterases forming DCFH, which was in non-fluorescent form and was oxidized to fluorescent compound DCF by ROS. Following exposure to 20% or 3% O_2_ for 1–5 days, cells were loaded with 10 μM DCFH-DA for 15 min at 37 °C and viewed under the fluorescence microscope. ROS measurement was carried out on FACS flow cytometer (Beckman Coulter, Fullerton, CA, U.S.).

### 3.5. Flow Cytometric Analysis

Fluorescence-activated cell sorter (FACS) analysis was performed to determine the monocyte surface expression of TLR10. Following exposure to 20% or 3% O_2_ for 3 days or 100 μM H_2_O_2_ for 0 to 4 h at 20% O_2_, THP-1 cells were harvested and washed twice, and stained with PE-conjugated anti-human TLR10 mAb (IMG-386D, Imgenex, San Diego, CA, U.S.) for 30 min at 4 °C in PBS. Cells were washed and resuspended in PBS, and analyzed on a FACS flow cytometer (Beckman Coulter).

### 3.6. Prediction of TF Binding Sites

To identify putative binding sites for NF-κB, AP-1, NF-AT and HIF-1 within the TLR10 promoter region, the TF binding site prediction program [39] Alggen Promo software, V3.0.2 (http://alggen.lsi.upc.es) was used with the default setting of 15% dissimilarity value.

### 3.7. Luciferase Reporter Gene Assay

THP-1 (1 × 10^6^) suspended in 10 mL RPMI-1640 medium were seeded in 25 cm^2^ T-flask. After incubating at 37 °C for 24 h, the cells were transiently transfected with 1 μg of luciferase reporter plasmids (pNF-κB-Luc, pAP-1-Luc or pNF-AT-luc) and 1 μg of pCMV-βGal using Nucleofector technology (Nucleofector II, Amaxa, Cologne, Germany). Cells were resuspended in 100 μL Nucleofector Solution V (Amaxa) and nucleofected with plasmids using program V-001. Following nucleofection, the cells were incubated in prewarmed RPMI 1640 medium for 4 h and then fresh complete medium was added. After 24 h, the cells were harvested, seeded in 24-well plates at a density of 1 × 10^5^ cells/well and treated with 100 μM H_2_O_2_ (Sigma Aldrich Corp., Sat. Louis, Mo, U.S.) for 6 h. For a positive control, cells were treated with 20 ng/mL of TNF-α (for NF-κB, eBioscience, San Diego, CA, U.S.), 20 ng/mL of PMA (for AP-1, Sigma), or 10 ng/mL of PMA and 1 μg/mL of ionomycin (for NF-AT, Sigma) for 6 h. After the stimulation, cells were harvested, washed and used for luciferase assay. Luciferase activity and β-galactosidase activity were assayed by using the luciferase and β-galactosidase enzyme assay system (Promega, Madison, WI, U.S.). The luminescence was quantitated as a relative light unit on a luminometer (Sirius luminometer, Berthold detection system GmbH, Germany). The results are expressed as relative reporter gene activity compared with controls after normalizing β-galactosidase activity.

### 3.8. Statistical Analysis

Student’s t tests were performed using statistical software SPSS 12.0. All data are mean ± SD from at least three different experiments. *P* < 0.05 was considered statistically significant.

## 4. Conclusions

Our results suggest that hypoxia or ROS can increase TLR10 expression in human monocytes and the transcriptional activities of NF-κB are mainly involved in this increase. Therefore, it is suggested that ROS, which can be produced by various exogenous stimuli, may play a crucial role in the regulation of expression and function of TLR10 as second messengers.

## Figures and Tables

**Table 1:** Effects of ROS on activities of TFs, NF-κB, AP-1 and NF-AT. The data are expressed as fold change in luciferase activity of cells treated with 100 μM H_2_O_2_ *versus* untreated cells. Cells at a density of 1 × 10^5^ cells/well were treated with 100 μM H_2_O_2_ for 6 h. For positive control, cells were treated with 20 ng/mL of TNF-α (for NF-κB), 20 ng/mL of PMA (for AP-1), or 10 ng/mL of PMA and 1 μg/mL of ionomycin (for NF-AT) for 6 h. After the stimulation, harvested cells were used for luciferase assay as described in Materials and Methods.

Transcription Factors	Relative Luciferase Activity (Fold)	
	ROS Treated	Positive Control
NF- κB	1.46 ± 0.45*	5.12 ± 1.52***
AP-1	1.32 ± 0.26**	18.37 ± 3.79***
NF-AT	1.21 ± 0.13***	3.04 ± 0.90***

* *P <* 0.05,

** *P <* 0.01,

*** *P <* 0.005.
